# Pharmacokinetics of Carboplatin in Combination with Low-Dose Cyclophosphamide in Female Dogs with Mammary Carcinoma

**DOI:** 10.3390/ani12223109

**Published:** 2022-11-10

**Authors:** Marília Carneiro Machado, Priscila Akemi Yamamoto, Leandro Francisco Pippa, Natália Valadares de Moraes, Fabiane Maria Fernandes Neves, Ricardo Dias Portela, Stella Maria Barrouin-Melo, Anna Hielm-Björkman, Ana Leonor Pardo Campos Godoy, Alessandra Estrela-Lima

**Affiliations:** 1 Department of Veterinary Anatomy, Pathology and Clinics, School of Veterinary Medicine and Zootechny, UFBA, Salvador 40170-110, BA, Brazil; 2 Department of Pharmaceutics, College of Pharmacy, University of Florida, Orlando, FL 32827, USA; 3 School of Pharmaceutical Sciences of Ribeirão Preto, University of São Paulo, Ribeirão Preto 14040-903, SP, Brazil; 4 Pharmacokinetics Laboratory, School of Pharmacy, Federal University of Bahia, Salvador 40170-115, BA, Brazil; 5 Laboratory of Immunology and Molecular Biology, Institute of Health Sciences, Federal University of Bahia, Salvador 40231-300, BA, Brazil; 6 Department of Equine and Small Animal Medicine, Faculty of Veterinary Medicine, University of Helsinki, P.O. Box 57, 00014 Helsinki, Finland; 7 Research Center on Mammary Oncology NPqOM/HOSPMEV, Federal University of Bahia, Salvador 40170-110, BA, Brazil

**Keywords:** breast cancer, carboplatin, dog, metronomic chemotherapy, pharmacokinetic parameters, survival rate

## Abstract

**Simple Summary:**

This study was designed to assess the effect of metronomic cyclophosphamide on carboplatin’s tolerability, efficacy, and pharmacokinetics in female dogs with mammary carcinoma. Sixteen female dogs with mammary carcinoma were treated with 300 mg/m^2^ intravenous (i.v.) carboplatin therapy (*n* = 8) or 300 mg/m^2^ i.v. carboplatin which was associated with 12.5 mg/m^2^/day oral cyclophosphamide (*n* = 8). A non-compartmental analysis was applied to calculate the PK parameters of carboplatin in the second and fourth chemotherapy cycles. The carboplatin pharmacokinetics showed high interindividual variability with 10-fold variation in the area under the plasma concentration–time curve (AUC) in the animals receiving carboplatin only. The systemic plasma exposure (AUC and C_max_) to carboplatin was equivalent in both of the treatments (carboplatin alone and carboplatin + cyclophosphamide). Carboplatin + metronomic cyclophosphamide was well-tolerated by all of the animals. Our results demonstrated that adding low daily doses of cyclophosphamide to the carboplatin therapy increased the survival rate of the female dogs with mammary cancer.

**Abstract:**

This prospective study aimed to evaluate the effect of metronomic cyclophosphamide on carboplatin’s tolerability, efficacy, and pharmacokinetics in dogs with mammary carcinoma. Sixteen female dogs with mammary carcinoma were divided into groups: 300 mg/m^2^ intravenous (i.v.) carboplatin therapy (G1 = 8) or 300 mg/m^2^ i.v. carboplatin which was associated with 12.5 mg/m^2^ oral cyclophosphamide in a metronomic regimen (G2 = 8). The investigated animals underwent a clinical evaluation, a mastectomy, a carboplatin chemotherapy, and serial blood sampling for the pharmacokinetic analysis. The adverse events and survival rates were monitored. A non-compartmental analysis was applied to calculate the pharmacokinetic parameters of carboplatin in the 2nd and 4th chemotherapy cycles. Carboplatin PK showed high interindividual variability with a 10-fold variation in the area under the plasma concentration–time curve (AUC) in G1. The systemic plasma exposure to carboplatin was equivalent in both of the treatments considering the AUC and maximum plasma concentration (C_max_) values. Although the red blood cells (*p* < 0.0001), platelets (*p* = 0.0005), total leukocytes (*p* = 0.0002), and segmented neutrophils (*p* = 0.0007) were reduced in G2, the survival rate increased (*p* = 0.0044) when it was compared to G1. In conclusion, adding low daily doses of cyclophosphamide to a carboplatin therapy showed promising outcomes in female dogs with mammary tumors.

## 1. Introduction

Mammary tumors account for 42% of all diagnosed tumors and 82% of reproductive tumors in female dogs according to the Veterinary Society of Surgical Oncology (VSSO) [1]. Due to the lack of effective adjuvant chemotherapy protocols for malignant or metastatic mammary tumors, antineoplastic agents can be combined to attack as many neoplastic cells as possible [2,3]. Doxorubicin, cyclophosphamide, 5-fluorouracil, cisplatin, and carboplatin are commonly used in maximally tolerated doses to prevent micrometastasis and solid tumor relapses [4]. Most chemotherapy drugs act by regulating cell division or damaging the DNA. Normal cells cannot be distinguished from malignant cells by chemotherapy. Thus, the tissues with rapidly dividing cells, mainly the bone marrow and gastrointestinal epithelial cells, are particularly sensitive to chemotherapy [2]. Because no study with maximum tolerated dose chemotherapy has proven to have efficacy on median the survival time for dog with carcinomas, the development of new therapeutic strategies with reduced toxicity and better efficacy is a significant challenge in cancer therapy.

In conventional chemotherapy, drug toxicity that is associated with high doses requires a rest period for the normal tissues to recover with increased cell proliferation rates [5]. In contrast, the metronomic administration of cytotoxic drugs involves a continuous or more frequent schedule at a much lower dose, thereby reducing the risk of adverse drug reactions [6]. Metronomic chemotherapy is emerging as a promising alternative that results in fewer adverse reactions and lower stress levels [6]. However, in veterinary medicine, there are still few studies related to its effectiveness, whether it is isolated or associated with conventional chemotherapy, especially in the case of mammary tumors in female dogs [7,8,9,10]. Nevertheless, the clinical implementation of new treatment combinations with low-dose chemotherapy requires studies to select effective treatment combinations for each tumor type and population [8,11,12,13].

Carboplatin is an alkyl anticancer medicine that was developed for human medicine. It is one of the drugs that are used for the adjuvant treatment of mammary neoplasm in female dogs, resulting in a significant increase in the survival rates and quality of life, mainly, when it is associated with naltrexone in low doses or cyclooxygenase inhibitors [7,14]. Carboplatin is less nephrotoxic than cisplatin is, and vomiting and diarrhea are less frequent and intense during a carboplatin treatment [15]. We aimed to investigate the effect of metronomic cyclophosphamide on the efficacy and tolerability of conventional carboplatin therapy in canine mammary cancer. Female dogs with mammary carcinomas were treated with 300 mg/m^2^ intravenous (i.v.) carboplatin or with i.v. carboplatin which was combined with an oral daily treatment of 12.5 mg/m^2^ cyclophosphamide. The combined therapy was assessed in carboplatin PK in terms of the plasma, disease progression, survival rate, and adverse drug reactions.

## 2. Materials and Methods

### 2.1. Animals

Female dogs with mammary carcinoma were recruited according to the following inclusion criteria: (a) the presence of a tumor larger than three centimeters; (b) clinical staging IV or mammary carcinoma with grade histopathological II or III; (c) having no other concomitant disease at the time of the assessment. The local Ethics Commission for the Use of Experimental Animals approved this research protocol, and all of the procedures were conducted according to the Brazilian College of Animal Experimentation (COBEA). All of the animal owners provided informed consent. The animals were prospectively recruited in a controlled, randomized clinical trial and allocated into groups considering the tumor size, clinic stage, diagnosis, and histological grades: a conventional i.v. carboplatin chemotherapy (300 mg/m^2^) group (G1), and a group with carboplatin chemotherapy which was combined with the oral administration of 12.5 mg/m^2^/day cyclophosphamide (G2).

### 2.2. Clinical Evaluation and Mastectomy

All of the animals were submitted to a clinical assessment, including a cancer survey, three-view thoracic radiography, a total abdomen ultrasound to assess the metastasis, and blood tests including a complete blood count, serum urea, creatinine, alkaline phosphatase, alanine aminotransferase, ionized calcium, and glucose concentrations. Malignant tumors were classified according to the TNM staging system, including the tumor size (T), the involvement of the regional lymph nodes (N), and the presence or absence of distant metastasis (M) [16]. All of the animals underwent a total unilateral mastectomy to remove the inguinal lymph node. The tumor samples were classified according to the World Health Organization (WHO) criteria, as described by Cassali et al. [4].

### 2.3. Chemotherapy Treatment

Twenty days after the surgery, the carboplatin chemotherapy was initiated in six sessions with an interval of twenty-one days between the sessions. The dogs received an intravenous 5 min infusion of carboplatin (300 mg/m^2^) through the cephalic vein. Only the animals in G2 orally received 12.5 mg/m^2^/day cyclophosphamide for eight months after starting the carboplatin chemotherapy. The cyclophosphamide tablet was administered approximately two hours after the first carboplatin session and daily for eight months in the morning. The blood samples were collected in the 2nd and 4th chemotherapy cycles before the carboplatin dosing and after 5, 15, 30, 45, 60, 120, and 240 min to evaluate the carboplatin PK. The blood samples were centrifuged at 3000× *g* for 5 min. The plasma samples were separated in aliquots and stored in cryotubes at −80 °C until the analysis.

Fourteen days after the carboplatin administration (nadir) and approximately 48 h before each session, the biological samples were collected for the urinalysis, total blood count, and serum biochemistry analysis. The laboratory tests attested to the condition of the animals to perform the chemotherapeutic carboplatin-based procedure. Ondansetron (0.2 mg/kg), ranitidine (2 mg/kg), and promethazine (0.1 mg/kg) were administered subcutaneously. All of the animals in all of the groups received these agents using this protocol. These drugs were prescribed orally for three consecutive days after the chemotherapy, except for promethazine, which was prescribed as a single dose.

### 2.4. Chemotherapy Adverse Reactions

All of the animals were screened weekly by phone calls during the chemotherapy and evaluated every fifteen days through a clinical exam focusing on the potential adverse drug reactions. For the evaluation of the adverse reactions caused by the chemotherapy protocol, we followed the criteria that were established by the Veterinary Cooperative Oncology Group-Common terminology criteria for adverse events (VCOG-CTCAE) [17]. This consensus recommends a grading scale (from 1 to 5) for the intensity of the adverse reactions caused by the chemotherapy (vomiting and diarrhea) in dogs and cats based on the following distinctions: grade 1 (mild): asymptomatic or mild symptoms, only diagnostic observations, no indication of therapeutic intervention; grade 2 (moderate): moderate limitation of daily activities, requires minimal or noninvasive outpatient intervention; grade 3 (severe or clinically significant, but not immediately fatal): significant limitation in daily activities, hospitalization is indicated; grade 4 (life-threatening): requires urgent therapeutic intervention; grade 5: death related to adverse drug reactions [17]. Dog owners provided the information by phone and filled out weekly reports indicating the possible adverse drug reactions. The Wilcoxon signed-rank test was performed to compare the adverse reaction scores in pairs between the groups in each chemotherapy session according to the chemotherapy protocol for female dogs with mammary carcinoma.

### 2.5. Analysis of Carboplatin in Dog Plasma

The carboplatin concentrations in the plasma were quantified by a fully validated high-performance liquid chromatography (HPLC) with UV detection at 229 nm. Chromatographic analysis was performed using a C18 reverse-phase column (250 × 4.6 mm). The mobile phase consisted of a mixture of 0.1 M potassium dihydrogen phosphate and 1 mM dipotassium edetate pH 3 (A) and acetonitrile (B). The mobile phase composition started with 100% A for 2 min, 95% A and 5% B from 2 to 10 min, and 100% A from 10 to 11 min.

The plasma samples (150 µL) were prepared by protein precipitation using 200 µL of acetonitrile. The samples were shaken for 15 min and centrifuged at 15,000× *g* for 15 min. After evaporating the supernatants until they dried up, the residue was reconstituted in 100 µL of water. An aliquot of 20 µL was injected into the HPLC system. The method was linear in the range of 1–75 µg/mL of the plasma, and the lower limit of quantification (LLOQ) was 1 µg/mL. The within-run precision, which was assessed as the relative standard deviation (RSD) for 5 sample replicates, was within 10.75% for the LLOQ and low (3 µg/mL), medium (40 µg/mL), and high (60 µg/mL) quality control levels. For the between-run precision, all of the quality control levels showed an RSD of up to 13.84%. The within- and between-run accuracy, which were assessed as the relative error (RE) for 5 replicates, were within ±15%, except for the LLOQ which had an RE of up to ±20%. Carboplatin was stable in the canine plasma samples for several stability assays such as the short-term stability (4 h at 24 °C), the long-term stability (70 days at −70 °C), and the post-processing ones (42 h at 18 °C), and up to 3 freeze/thaw cycles (room temperature/−70 °C) (Supplementary Material, Table S1).

### 2.6. PK Analysis of Carboplatin in Plasma

A non-compartmental analysis (NCA) was performed using Phoenix 64 WinNonlin software v.8.3.4.295 (Certara, Princeton, NJ, USA). The terminal slope of the plasma concentration–time profile was selected to derive the elimination half-life (λz). The area under the plasma concentration–time curve was calculated from the time of the dosing to the last observation time (AUC_0–t_) using the linear/log trapezoidal method. AUC_0–∞_ was calculated by adding AUC_0–t_ to the ratio of the final measured concentration (C_last_)/λ_z_. The PK parameters’ mean residence time (MRT), half-life (t_1/2_), total clearance (CL), and the volume of distribution were estimated according to the standard NCA equations. If the percentage of the AUC-extrapolated area was greater than 20%, the total AUC_0–∞_ was considered to be unreliable. Paired and unpaired *t*-tests of the log-transformed data were used to compare the pharmacokinetic parameters between the phases and groups, respectively, once the normality was assessed using the Shapiro–Wilk test. *p* values that were lower than 0.05 were considered to be significant. The descriptive statistics for the PK parameters in each group were shown as the geometric means and geometric coefficients of the variation. If the geometric means ratio had a 90% confidence interval (90% CI) that was within the interval of 0.8–1.25, the treatments were considered to be equivalent [18]. The statistical analysis was conducted using R software v.4.3.1 [19], and the plots were generated using the ggplot2 package v.3.3.6 [20].

### 2.7. Follow-Up and Survival Rate

The clinical and laboratory tests were performed every 15 days. The thoracic radiological examinations and the total abdominal ultrasounds were performed monthly. One month after the completion of the conventional carboplatin chemotherapy cycle with carboplatin (six sessions) and at the end of metronomic chemotherapy with cyclophosphamide (eight months), the animals were evaluated clinically evaluated by laboratory tests, and their neutrophil, platelet, and red cell parameters were within the normal range. This follow-up was maintained until the end of the experiment or the animal’s death. The overall survival time was defined as the period (in days) between the surgical excision of the primary tumor and the date of death due to the disease or the date of completion of the study. The overall survival of the animals was verified by phone calls at the end of the experiment.

All of the animals were screened weekly during the chemotherapy and monthly after the end of chemotherapy until the end of the study. The medical team assessed the need for humanitarian euthanasia and carried it out with the owners’ consent [21]. The patients who died during the follow-up period were necropsied to determine the cause of the death and to identify the possible metastases or chemotherapy-induced lesions. The survival and disease-free interval curves were estimated by the Kaplan–Meier method and compared by the Log-rank (Mantel–Cox) or Cox test in a univariate or multivariate analysis, respectively. The analyses were performed using Prism software v5.0 (GraphPad, San Diego, CA, USA). For all of the evaluations, the statistical significance was set at 5% (*p* < 0.05).

## 3. Results

### 3.1. Clinical and Pathological Features

From 50 eligible animals with mammary tumors, 16 female dogs of mixed breeds were included in the current investigation. The presence of other types of tumors, in situ mammary carcinoma, concomitant diseases at the time of the assessment and their non-attendance on the day of the chemotherapy procedure were considered to be the exclusion criteria. The included female dogs were aged from seven to fifteen years old, and they weighed from 7 to 15 kg. Despite the different histological types of mammary tumors, a homogenous distribution was observed between the groups (Table 1). Invasive areas were used for the histopathological grading of the tumors, with fourteen tumors being classified as grade II and two tumors being classified as grade III. According to the clinical staging evaluation, the dogs were classified as stages II, III, or IV. The female dogs that died presented confirmed metastatic foci after necropsy, and the histopathological evaluation of the organs were reclassified to stage V due to a worse prognosis, regardless of the regional metastasis.

### 3.2. Adverse Effects

**Figure 1:**
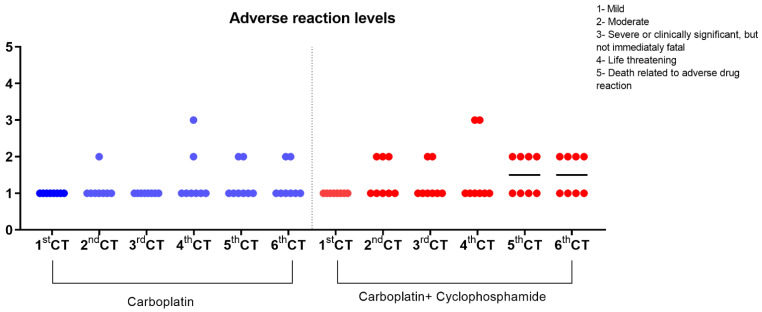
Comparison of adverse reaction levels in each chemotherapy (CT) session according to the chemotherapy protocol in female dogs with mammary carcinoma. The classification herein follows the one that has been described by the VCOG [15]. The Wilcoxon signed-rank test was performed in pairs and showed no significant difference (*p* < 0.05) between groups in all chemotherapy sessions.

**Figure 2:**
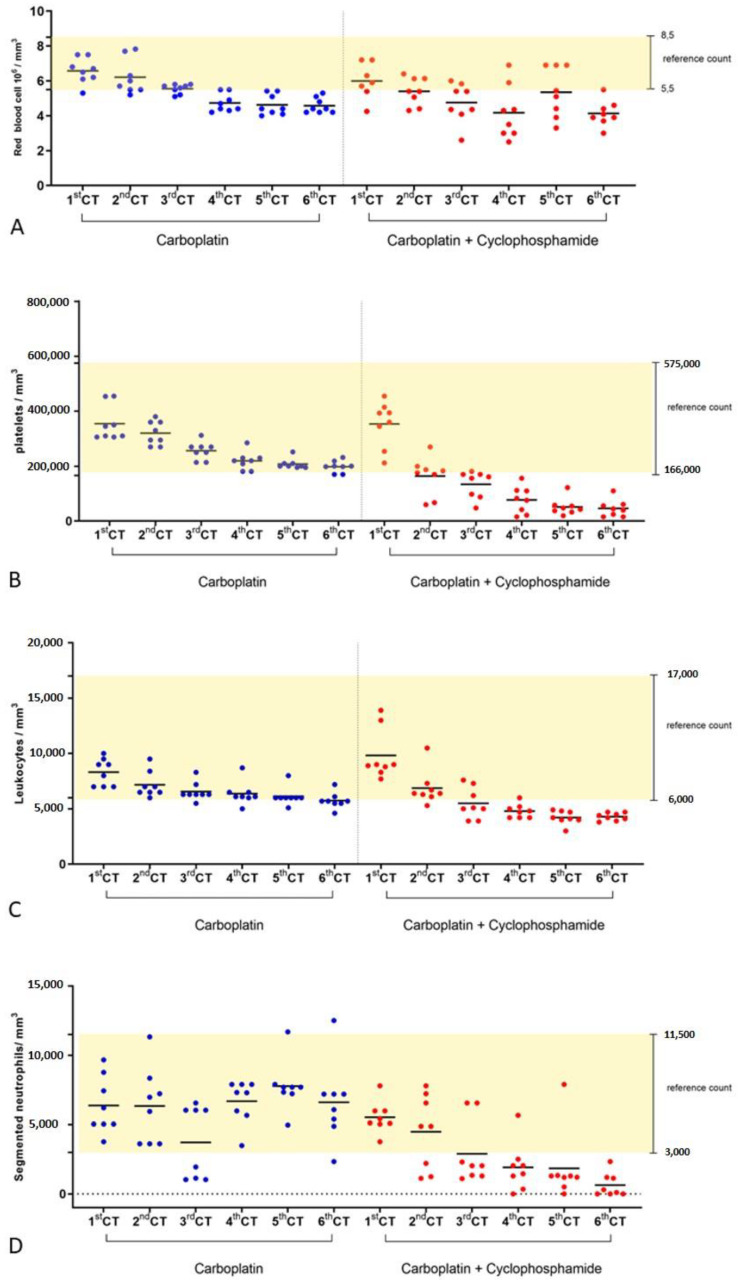
Comparing hematological biomarkers of therapeutic toxicity in chemotherapy (CT) with carboplatin (G1) or carboplatin + cyclophosphamide treatments (G2). (**A**) Total erythrocytes, (**B**) platelets, (**C**) total leukocytes, and (**D**) neutrophil granulocytes. Approximately 48 h before the beginning of chemotherapy, biological samples were collected for urine analysis, total blood count, and serum biochemical analysis. In 1st CT, these values were considered the baseline for all analyzes. The Wilcoxon signed-rank test was performed in pairs and showed significant differences between the groups for all hematological biomarkers, with *p* < 0.05.

Regarding chemotherapy, 81.25% (13/16) of the female dogs completed the six sessions with intervals of 21 days between them, as predicted in the established protocol. A total average time of up to five days was observed for the recovery of the hematological parameters, regardless of the treated group. However, for the animals that presented more significant hematological changes during the treatment, the time required for hematological recovery was, on average, seven days for the carboplatin group and ten days for the carboplatin + cyclophosphamide group. In these cases, for two animals of the metronomic group and one animal of the carboplatin treatment group, we needed to increase the interval between the sessions to 28 days with the objective to reach a complete clinical recovery. Among the adverse events of the treatment, 18.75% (3/16) of the female dogs presented vomiting up to two days after the chemotherapy session and in the drug nadir, and 12.5% (2/16) of them showed diarrhea, mainly in G2. Regardless of the chemotherapy session and treatment group allocation, most of the evaluated dogs did not show adverse drug reactions or had mild reactions without the indication of an intervention occurring. There was no difference in the degree of adverse reactions between the groups (Figure 1; Table 2). The major hematological adverse drug events included anemia, thrombocytopenia, and neutropenia. Thrombocytopenia was the most critical finding in both of the groups. A marked reduction in the red blood cells (*p* < 0.0001) and platelets (*p* = 0.0005) was observed in G2 when it was compared to G1 (Figure 2A,B). Neutropenia was observed in both groups, being more frequent in G2, with a lower numbers of total leukocytes (*p* = 0.0002) and segmented neutrophils (*p* = 0.0007) (Figure 2C,D). No significant differences were observed between the experimental groups in the clinical biochemistry and urinalysis parameters. No animal that underwent the treatment with cyclophosphamide presented with cystitis.

### 3.3. Pharmacokinetic Analysis of Carboplatin

**Figure 3:**
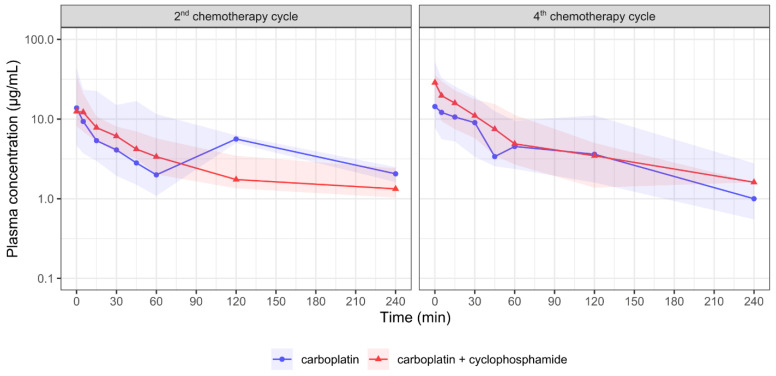
Carboplatin pharmacokinetic (PK) profiles in female dogs with mammary carcinoma in the second and fourth chemotherapy cycles. Female dogs were treated with 5 min intravenous (i.v.) infusion of 300 mg/m^2^ carboplatin in six chemotherapy sessions with twenty-one-day intervals between sessions without (blue) and with (red) association to 12.5 mg/m^2^/day oral cyclophosphamide in a metronomic regimen. PK profiles are shown as solid lines for median values and shaded areas for 5th–95th percentiles.

A method for the analysis of carboplatin in the canine plasma by HPLC was developed and validated. The method showed suitable linearity, precision, accuracy, stability, and confidence limits and was successfully applied to determine the plasma concentrations of carboplatin in the dogs (Table S1). Two animals from G1 were excluded from the statistical analysis because they did not meet the criteria of the extrapolated AUC area being lower than 20%. The data were considered to be normally distributed after the log-transformation (Supplemental Table S2), and they are presented as boxplots (Figure S1). The carboplatin PK showed high interindividual variability. A 10-fold variation in the AUC was observed in the animals that were treated with carboplatin only. The geometric mean AUC_0–∞_ were 525.1 and 941.7 µg⋅min/mL at the 2nd and 4th cycles, respectively (Table 3, Figure 3). The CL values were 34.3 and 19.1 L/h/m^2^ in the 2nd and 4th cycles, respectively, in the animals receiving carboplatin only. The animals receiving carboplatin and cyclophosphamide showed a CL of 25.8 in the 2nd cycle and 18.3 L/h/m^2^ in the 4th cycle (Table 3). No differences were observed in the other PK parameters. The systemic plasma exposure to carboplatin was considered to be equivalent in both of the treatments. The dogs that were treated with the combined therapy showed carboplatin AUC_0–∞_ values of 698.4 and 984.2 µg⋅min/mL in the 2nd and 4th doses, respectively (Table 3). When we were comparing the 2nd with the 4th cycle, the C_max_ of carboplatin increased in the 4th cycle only in the animals receiving the carboplatin + cyclophosphamide protocol. The AUC_0-t_ and AUC_0–∞_ were similar across the 2nd and 4th cycles for both of the groups (Supplementary Material, Table S3).

### 3.4. Survival Time

**Figure 4:**
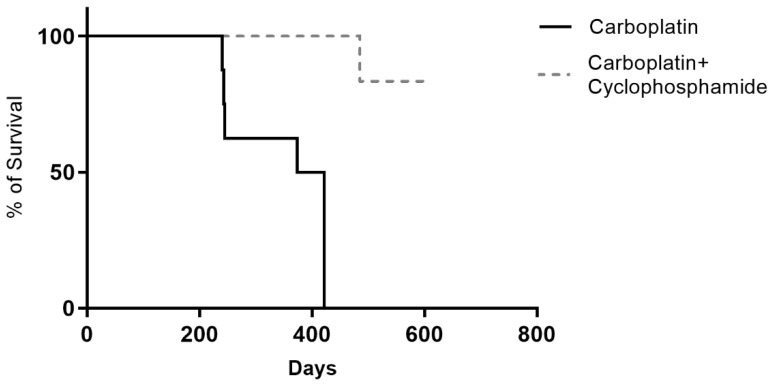
Survival curve of female dogs after carboplatin-associated treatment (dashed line) or not (solid line) with metronomic cyclophosphamide.

The minimum survival time that was observed was 240 days for a female dog receiving carboplatin only, who died due to lung metastasis, which was confirmed by a histopathological evaluation. The maximum survival time was 613 days after the mastectomy, which was attributed to a dog from the group that received carboplatin + cyclophosphamide and who is still being monitored. The median survival rate in the carboplatin-treated group (G1) was 398 days, while that of the carboplatin + cyclophosphamide group (G2) did not reach the median value (Figure 4). There were significant differences between the carboplatin + cyclophosphamide group (G2) survival curves and the carboplatin group (G1). A higher number of deaths were observed in G1 due to the progression of the cancer (metastasis) (*n* = 5/8; 62.5%, *p* = 0.0117, HR 10.06 and IC 95% 1.839–54.99) (Figure 4). After necropsy, the most frequent distant metastasis sites observed were the lungs, skin, and liver, and the principal cause of mortis was acute respiratory failure. In G2, only one death was detected due to advanced heart failure which was unrelated to the chemotherapy. No animal was censored as it was possible to monitor all of the animals during the study period.

## 4. Discussion

Due to their high incidence in female dogs, mammary carcinomas require safe and effective multimodal pharmacological strategies. Our data show that adding low doses of oral cyclophosphamide to a carboplatin treatment significantly reduces the values of the hematological parameters. However, these effects were not enough to trigger clinical outcomes or impact the dogs’ quality of life. Similar results were observed in the dogs with osteosarcoma that were treated with metronomic cyclophosphamide, presenting mild laboratory alterations without there being any associated clinical findings [13].

Adverse drug reactions of carboplatin include renal alterations, gastrointestinal effects (such as anorexia, emesis, and constipation), and liver toxicity [7]. The renal and hepatic functions of the female dogs in both of the groups were not affected by the chemotherapy throughout the study period. No changes in the serum biochemistry parameters—creatinine, urea, and alanine aminotransferase—were reported in the female dogs with mammary tumors receiving doxorubicin and cyclophosphamide [7]. Myelosuppression, which is mainly characterized by neutropenia and thrombocytopenia, was reported approximately from 14 to 21 days after the carboplatin treatment [22]. Coffee and colleagues [23] described a relationship between decreasing weight and the risk of chemotherapy-induced myelosuppression. In the current investigation, this relationship was not observed as the hematological parameters did not change according to the body weight of the female dogs.

Increased survival was observed for carboplatin + cyclophosphamide-treated dogs, regardless of the presence or absence of metastasis. Similarly, the female dogs that were treated with carboplatin + metronomic thalidomide and cyclophosphamide showed prolonged survival when they were compared to those in the carboplatin + cyclophosphamide group [24]. Five female dogs that were treated only with carboplatin died due to the disease’s progression (5/16). These animals were necropsied, and the distant metastases in the lung, skin, and liver were observed. Acute respiratory failure was identified as the cause of death. Considering the cause of death and the presence of late metastases, similar results were described by Costa-Santos et al. [25]. In the current study, the histological analyses of the affected organs confirmed the presence of metastases.

This study describes for the first time the effect of metronomic cyclophosphamide on the kinetic disposition of carboplatin in dogs. A previous study investigated drug toxicity in combined metronomic therapy and maximal-tolerated dose chemotherapy in dogs with osteosarcoma, but the authors did not report the carboplatin PK data [8]. Another study investigating the effectiveness of metronomic cyclophosphamide with adjuvant meloxicam in dogs with appendicular osteosarcoma following a limb amputation and carboplatin chemotherapy did not report on the carboplatin PK [13]. Metronomic chemotherapy provides a lower metastasis index and reduces the adverse drug reactions, thus improving the dogs’ quality of life and well-being [26,27].

Previous studies have reported carboplatin PK in dogs that were treated with a dose range of 30–580 mg/m^2^ [15,22,28,29]. Carboplatin presents a linear PK when it is administered in therapeutic doses to dogs [28]. It does not bind immediately and reversibly to mouse, dog, or human plasma proteins [30]. Unlike cisplatin, carboplatin binds slowly to the plasma proteins (24–50%) [28,31]. Renal excretion is the main pathway of carboplatin elimination in humans (57–82%) and dogs (46–70%) [32,33,34]. Carboplatin clearance is highly correlated with the glomerular filtration rate in humans [32]. Our results show that the carboplatin PK is not affected by the coadministration of low doses of cyclophosphamide in female dogs with mammary carcinoma. The animals that were treated with carboplatin + cyclophosphamide showed equivalent systemic exposure results, which were assessed by the plasma C_max_ and AUC values in the 2nd and 4th chemotherapy cycles. The total systemic exposure (AUC) to carboplatin was similar across the 2nd and 4th cycles (Table S3). The carboplatin C_max_ increased in the 4th cycle only in the animals receiving carboplatin + cyclophosphamide. Though the cyclophosphamide PK parameters were not explored, we can anticipate that the increased carboplatin C_max_ may be related to the cumulative effect that is had on the blood count parameters over time.

Combined chemotherapy treatments increase the survival of animals with neoplasms as they have different action mechanisms and reach a larger number of neoplastic cells, thereby increasing the effectiveness of the treatment. The mechanism of action of carboplatin occurs by connecting within and between the DNA chains and inhibits protein synthesis in a non-specific cell cycle phase [35]. Carboplatin is activated intracellularly to react with the platinum complexes that bind to nucleophilic groups, which will produce inter and intra-pilgrims in the DNA and inhibit their replication, RNA transcription, and protein synthesis. This results in apoptosis and the inhibition of the growth of the neoplastic cells [36].

Cyclophosphamide has a strong antiangiogenic and immunomodulatory capacity, and gradually, it contributes to control neoplasias [12]. When they are exposed to cyclophosphamide, the endothelial cells in the tumor vasculature are directly affected by the cytotoxic drugs [37,38,39] through a selective induction of apoptosis and the selective inhibition of proliferation and migration [40,41,42]. These cells are also indirectly affected by a modulation of the angiogenic factors—the upregulation of the anti-angiogenic factors (e.g., endostatin and thrombospondin-1 [TSP-1]) and the downregulation of the pro-angiogenic factors (e.g., vascular endothelial growth factor [VEGF], basic fibroblast growth factor [bFGF], hypoxia-inducible factor-1α [HIF-1α], and the angiopoeitin family) [2,39]. Among the immunomodulatory functions of cyclophosphamide, we can mention: the stimulation of interferon gamma, the increase in natural killer T lymphocytes, the maturation of dendritic cells, and increase in the lifetime of memory T cells. This drug also acts by decreasing the concentration of immunosuppressive cytokines, such as TGF-b, IL-10, and IL-2, and reducing the count of regulatory T lymphocytes [26]. Thus, the combination of the carboplatin mechanism that is associated with cyclophosphamide produces an antineoplastic effect by different mechanisms, cytotoxic, antiangiogenic, and immunomodulators, which result in the control of neoplastic growth progression and a more prolonged survival for the patient.

The clinical biochemistry and blood counts in the dogs that were treated with carboplatin + cyclophosphamide were not associated with adverse drug reactions or other clinical outcomes. Therefore, the carboplatin + metronomic cyclophosphamide therapy was superior to the carboplatin only one in the female dogs with regional lymph node metastasis or moderate/high-grade mammary carcinoma. However, studies evaluating the isolated cyclophosphamide effect on the metronomic regimen on dog survival with mammary carcinoma are still needed.

## 5. Conclusions

Our results showed that the systemic plasma exposure to carboplatin was equivalent in animals in both of the chemotherapy regimens. Considering the increased survival rate, carboplatin + metronomic cyclophosphamide is a complementary therapeutic alternative for female dogs with high-grade or metastatic mammary carcinomas. Further prospective, randomized, larger-scale clinical trials are required to evaluate this promising combination therapy.

## Figures and Tables

**Table 1:** Clinical and pathological characteristics of female dogs with mammary neoplasms. Sixteen animals were evaluated.

Case	Age Years	Body Condition	T	N	M	TNM Stage	Survival	Histological Types and Grade
Carboplatin								
1	11	lean	3–5 cm	N0	M0	II	240	Solid carcinoma Grade 2
2	8	normal	3–5 cm	N0	M0	II	240	Solid carcinoma Grade 2
3	14	normal	>5 cm	N0	M0	III	383	Papillary carcinoma Grade 2
4	12	normal	>5 cm	N0	M0	III	422	Mixed tumor carcinoma Grade 2
5	11	normal	>5 cm	N0	M0	III	374	Mixed tumor carcinoma Grade 2
6	15	obese	>5 cm	N1	M0	IV	245	Solid carcinoma Grade 3
7	10	normal	3–5 cm	N1	M0	IV	390	Mixed tumor carcinoma Grade 2
8	7	normal	>5 cm	N1	M0	IV	386	Mixed tumor carcinoma Grade 2
Carboplatin + Cyclophosphamide								
9	12	obese	>5 cm	N1	M0	IV	485	Solid carcinoma Grade 3
10	8	normal	3–5 cm	N1	M0	IV	600	Mixed tumor carcinoma Grade 2
11	14	normal	>5 cm	N0	M0	III	570	Mixed tumor carcinoma Grade 2
12	13	lean	>5 cm	N0	M0	III	517	Papillary carcinoma Grade 2
13	10	normal	>5 cm	N0	M0	III	360	Solid carcinoma Grade 2
14	12	normal	>5 cm	N0	M0	III	368	Solid carcinoma Grade 2
15	9	normal	>5 cm	N0	M0	III	557	Mixed tumor carcinoma Grade 2
16	12	normal	>5 cm	N1	M0	IV	613	Mixed tumor carcinoma Grade 2

T: tumor size; N: involvement of regional lymph nodes (N0: absence; N1: presence); M: presence or absence of distant metastases. Stages II, III, and IV were defined based on TNM system that has been previously described by Owen [14].

**Table 2:** There was vomiting and diarrhea in female dogs with mammary carcinoma after each chemotherapy (CT) session, according to the chemotherapy protocol. The numbers describe the number of vomiting and diarrhea events that each animal experienced from each group presented after 21 days of each chemotherapy session in carboplatin and carboplatin + cyclophosphamide treatment groups. Other adverse clinical events that have been described by the VCOG classification [15] were not significantly evident in these animals.

	Carboplatin (G1)						Carboplatin + Cyclophosphamide (G2)					
Case Nº	1st CT	2nd CT	3rd CT	4th CT	5th CT	6th CT	1st CT	2nd CT	3rd CT	4th CT	5th CT	6th CT
	Vomiting											
1	0	0	0	0	0	0	0	0	0	0	0	1
2	0	0	0	0	0	0	0	3	0	0	3	3
3	0	0	0	3	3	3	0	0	0	0	1	1
4	0	0	0	0	0	0	0	3	3	11	5	4
5	0	0	0	0	0	0	0	0	0	0	0	1
6	0	0	0	0	0	0	0	3	3	11	4	3
7	0	0	0	0	0	0	0	0	0	0	1	0
8	0	3	0	11	3	3	0	0	0	0	3	3
	Diarrhea											
1	0	0	0	0	0	0	0	0	0	2	0	0
2	0	0	0	0	0	0	0	0	0	0	0	0
3	0	0	0	0	0	0	0	0	0	0	0	0
4	0	0	0	0	0	0	0	0	0	0	0	0
5	0	0	0	0	0	0	0	0	0	0	0	0
6	0	0	0	0	0	0	0	0	0	0	0	0
7	0	0	0	0	0	0	0	0	0	0	1	0
8	0	0	0	0	0	0	0	0	0	0	0	0

**Table 3:** PK parameters of carboplatin in the presence and absence of cyclophosphamide.

Parameter	2nd Cycle				4th Cycle			
	Carboplatin	Carboplatin + Cyclophosphamide	GMR ^a^	*p*-Value	Carboplatin	Carboplatin + Cyclophosphamide	GMR ^a^	*p*-Value
AUC_0-t_ (µg/mL⋅min)	423.4 (176.7) [159.1–1127.0]	557.2 (53.5) [398.2–779.7]	1.32 [0.49–3.56]	0.61	754.0 (125.3) [339.1–1676.7]	831.1 (61.9) [567.4–1217.3]	1.10 [0.48–2.54]	0.83
AUC_0-∞_ (µg/mL⋅min)	525.1 (155.3) [211.1–1306.5]	698.4 (50.6) [507.2–961.5]	1.33 [0.53–3.36]	0.58	941.7 (99.6) [475.7–1864.2]	984.2 (56.2) [693.0–1397.7]	1.05 [0.51–2.15]	0.91
C_max_ (µg/mL)	12.9 (109.6) [6.2–26.9]	15.2 (53.3) [10.9–21.3]	1.18 [0.55–2.51]	0.70	17.7 (108.3) [8.6–36.4]	27.3 (33.0) [22.0–33.8]	1.55 [0.74–3.22]	0.29
CL (L/h/m^2^)	34.3 (155.3) [13.8–85.3]	25.8 (50.6) [18.7–35.5]	-	-	19.1 (99.6) [9.7–37.8]	18.3 (56.2) [12.9–26.0]	-	-
t_1/2_ (min)	39.1 (55.1) [26.8–57.1]	46.9 (81.4) [29.1–75.6]	-		49.5 (58.8) [31.6–77.5]	32.9 (45.8) [24.5–44.0]	-	-
MRT (min)	52.3 (52.4) [36.4–75.1]	61.8 (86.3) [37.5–101.9]	-		70.0 (58.4) [44.9–109.4]	44.3 (47.9) [32.7–60.1]	-	-
Vd_z_ (L/m^2^)	36.7 (78.9) [22–61.1]	29.4 (49.2) [21.6–40.2]	-		23.0 (111.9) [11.0–48.4]	14.5 (43.7) (11–19.2)	-	-
Vd_ss_ (L/m^2^)	34.0 (81.8) [20.1–57.5]	26.9 (53.7) [19.2–37.7]	-		22.6 (108) [11–46.7]	13.6 (45.4) [10.1–18.1]	-	-

Data are presented as geometric means (geometric percentage of coefficient of variation) at 90% confidence interval. GMR ^a^: geometric mean ratio (90% confidence interval). AUC_0–t_: area under the plasma concentration–time curve calculated from the time of dosing up to the last observation time. AUC_0–∞_: area under the plasma concentration–time curve from the time of dosing extrapolated to infinity. C_max_: maximum plasma concentration. CL: total clearance. MRT: mean residence time. Vd_z_: volume of distribution. Vd_ss_: volume of distribution at steady state.

## Data Availability

The corresponding author’s data supporting this study’s findings are available upon reasonable request.

## Supplementary Materials

The following supporting information can be downloaded at: https://www.mdpi.com/article/10.3390/ani12223109/s1, Table S1: Validation of the HPLC-UV method for analysis of carboplatin in canine plasma; Table S2: Shapiro–Wilk tests for raw and log-transformed values; Table S3: PK parameters of carboplatin in the 2nd and 4th chemotherapy cycles; Figure S1: Boxplots of the PK parameters of carboplatin in the presence and absence of cyclophosphamide.
