# Supplementary material for: Pharmacokinetics of Carboplatin in Combination with Low-Dose Cyclophosphamide in Female Dogs with Mammary Carcinoma
Source: Animals (Basel). 2022 Nov 10;12(22):3109. doi: 10.3390/ani12223109 (PMC9686876; doi:10.3390/ani12223109)
Supplement: Supplementary file 1 [file animals-12-03109-s001.zip › Supplementary Figure S1.pdf]

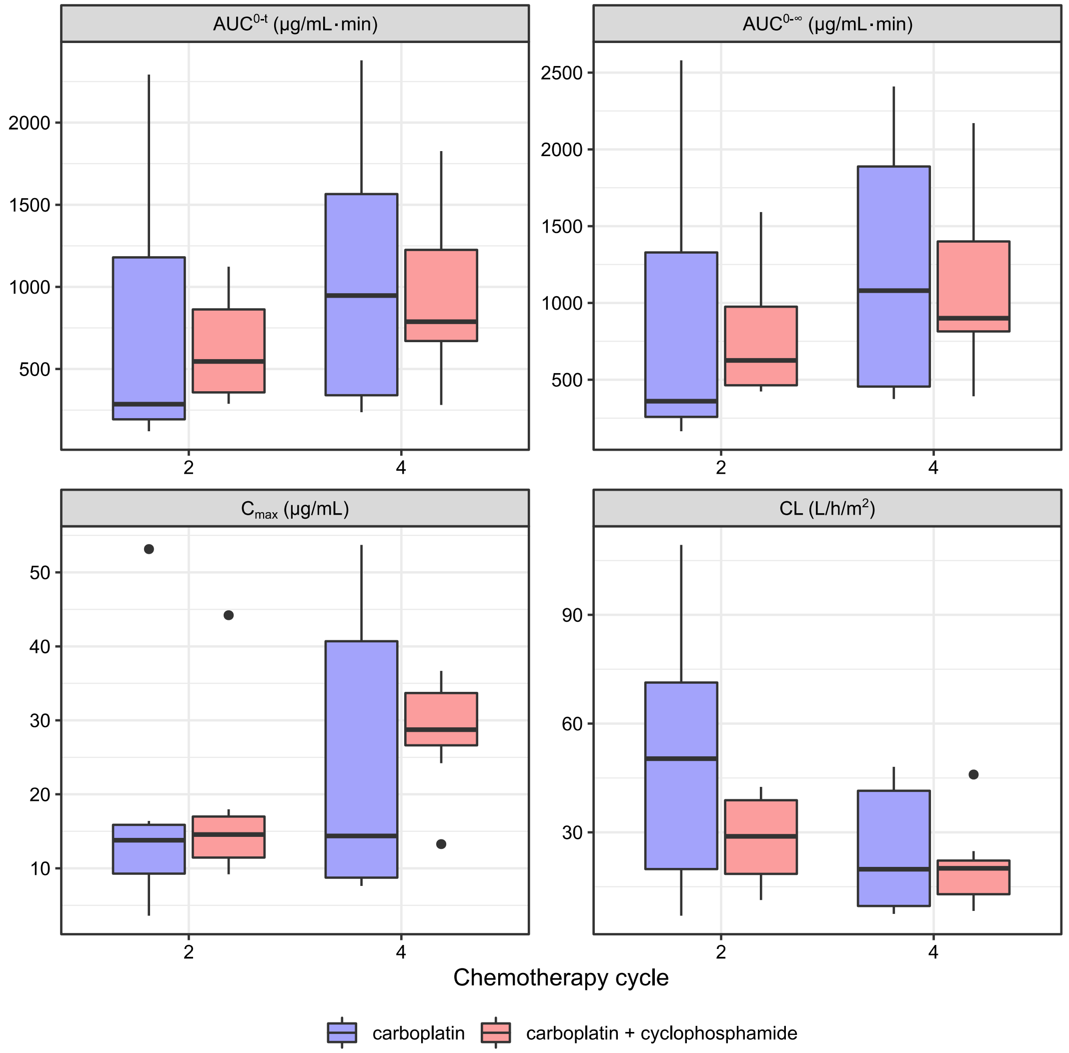

Figure S1. Boxplots of the PK parameters of carboplatin in the presence and absence of cyclophosphamide.
