# Supplementary material for: Pharmacokinetics of Carboplatin in Combination with Low-Dose Cyclophosphamide in Female Dogs with Mammary Carcinoma
Source: Animals (Basel). 2022 Nov 10;12(22):3109. doi: 10.3390/ani12223109 (PMC9686876; doi:10.3390/ani12223109)
Supplement: Supplementary file 1 [file animals-12-03109-s001.zip › Supplementary Table S1.pdf]

Table S1. Validation of the LC-UV method for analysis of carboplatin in canine plasma.

**Validation parameters**		
**Linearity**	1-75 µg/mL	
R	0.99713	
Slope	6144.74	
y-intercept	-6138.35	
Weight	$1/x^2$	
**Precision**	*Within-run (CV %, n=5)*	*Between-run (CV %, n=3)*
LLOQ (1 µg/mL)	7.67	9.21
LQC (3 µg/mL)	10.68	13.84
MQC (40 µg/mL)	10.75	11.64
HQC (60 µg/mL)	7.94	11.49
**Accuracy**	*Within-run (RE %, n=5)*	*Between-run (RE %, n=3)*
LLOQ (1 µg/mL)	13.15	18.57
LQC (3 µg/mL)	-5.98	-3.67
MQC (40 µg/mL)	0.50	0.32
HQC (60 µg/mL)	-4.89	0.42
**Stability (RE %, n=3)**		
Short-term (4 h at 24 ºC)		
LQC (3 µg/mL)	12.95	
HQC (60 µg/mL)	11.46	
Long-term (70 days at -70 ºC)		
LQC (3 µg/mL)	1.63	
HQC (60 µg/mL)	13.58	
Post-preparative (42 h at 18 ºC)		
LQC (3 µg/mL)	-10.75	
HQC (60 µg/mL)	-14.48	
Freeze/thaw cycles (n=3, -70 ºC)		
LQC (3 µg/mL)	-8.28	
HQC (60 µg/mL)	13.11	
Standard solution (30 days at 4-8 ºC)		
12.5 µg/mL	-5.98	
1000 µg/mL	-8.26	

r: linear correlation coefficient; CV: coefficient of variation [(standard deviation/mean concentration) x 100]; RE: relative error {[(observed mean concentration – nominal concentration)/nominal concentration] x 100}; LLOQ: lower limit of quantification; LQC: low quality control; MQC: medium quality control; HQC: high quality control.
