# Supplementary material for: Pharmacokinetics of Carboplatin in Combination with Low-Dose Cyclophosphamide in Female Dogs with Mammary Carcinoma
Source: Animals (Basel). 2022 Nov 10;12(22):3109. doi: 10.3390/ani12223109 (PMC9686876; doi:10.3390/ani12223109)
Supplement: Supplementary file 1 [file animals-12-03109-s001.zip › Supplementary Table S2.pdf]

Table S2. Shapiro-Wilk tests for raw and log-transformed values.

Variable	Raw values		Log-transformed values	
	**W**	***p*-value**	**W**	***p*-value**
$AUC^{0\text{-}t}$ (μg/mL·min)	0.88	0.0051 (**)	0.97	0.57
$AUC^{0\text{-}\infty}$ (μg/mL·min)	0.89	0.0060 (**)	0.97	0.50
C_{max} (μg/mL)	0.89	0.0062 (**)	0.97	0.52
CL (L/h/m^2)	0.83	0.00046 (***)	0.97	0.50

$p < 0.05$ means the distribution of the sample differs from a normal distribution. (**) $p < 0.01$, (**) $p < 0.001$.
