# Supplementary material for: Pharmacokinetics of Carboplatin in Combination with Low-Dose Cyclophosphamide in Female Dogs with Mammary Carcinoma
Source: Animals (Basel). 2022 Nov 10;12(22):3109. doi: 10.3390/ani12223109 (PMC9686876; doi:10.3390/ani12223109)
Supplement: Supplementary file 1 [file animals-12-03109-s001.zip › Supplementary Table S3.pdf]

Table S3. PK parameters of carboplatin in the 2nd and 4th chemotherapy cycles.

Parameter	Carboplatin				Carboplatin + Cyclophosphamide			
	2nd Cycle	4th Cycle	GMR[a]	p-Value	2nd Cycle	4th Cycle	GMR[a]	p-Value
**$AUC_{0\text{-}t}$ (μg/mL·min)**	423.4 (176.7) [159.1–1127.0]	754.0 (125.3) [339.1–1676.7]	1.78 [0.57–5.58]	0.38	557.2 (53.5) [398.2–779.7]	831.1 (61.9) [567.4–1217.3]	1.49 [0.93–2.39]	0.16
**$AUC_{0\text{-}\infty}$ (μg/mL·min)**	525.1 (155.3) [211.1–1306.5]	941.7 (99.6) [475.7–1864.2]	1.79 [0.64–5.04]	0.33	698.4 (50.6) [507.2–961.5]	984.2 (56.2) [693.0–1397.7]	1.41 [0.91–2.19]	0.19
**C_{max} (μg/mL)**	12.9 (109.6) [6.2–26.9]	17.7 (108.3) [8.6–36.4]	1.36 [0.54–3.44]	0.56	15.2 (53.3) [10.9–21.3]	27.3 (33.0) [22.0–33.8]	1.79 [1.23–2.60]	0.02 *

Data are presented as geometric means (geometric percentage of coefficient of variation) and [90% confidence interval]. GMR[a]: geometric mean ratio [90% confidence interval]. $AUC_{0\text{–}t}$: area under the plasma concentration-time curve calculated from the time of dosing up to the last observation time. $AUC_{0\text{–}\infty}$: area under the plasma concentration-time curve from the time of dosing extrapolated to infinity. C_{max}: maximum plasma concentration. (*) $p < 0.05$.
